# Diverging trajectories of neighborhood disadvantage by race and birth cohort from childhood through young adulthood

**DOI:** 10.1371/journal.pone.0283641

**Published:** 2023-04-19

**Authors:** Jennifer Candipan, Robert J. Sampson

**Affiliations:** 1 Department of Sociology, Brown University, Providence, RI, United States of America; 2 Department of Sociology, Harvard University, Cambridge, MA, United States of America; Federal University of Santa Maria: Universidade Federal de Santa Maria, BRAZIL

## Abstract

Prior research has established the greater exposure of African Americans from all income groups to disadvantaged environments compared to whites, but the traditional focus in studies of neighborhood stratification obscures heterogeneity within racial/ethnic groups in residential attainment over time. Also obscured are the moderating influences of broader social changes on the life-course and the experiences of Latinos, a large and growing presence in American cities. We address these issues by examining group-based trajectory models of residential neighborhood disadvantage among white, Black, and Latino individuals in a multi-cohort longitudinal research design of over 1,000 children from Chicago as they transitioned to adulthood over the last quarter century. We find considerable temporal consistency among white individuals compared to dynamic heterogeneity among nonwhite individuals in exposure to residential disadvantage, especially Black individuals and those born in the 1980s compared to the 1990s. Racial and cohort differences are not accounted for by early-life characteristics that predict long-term attainment. Inequalities by race in trajectories of neighborhood disadvantage are thus at once more stable and more dynamic than previous research suggests, and they are modified by broader social changes. These findings offer insights on the changing pathways by which neighborhood racial inequality is produced.

## Introduction

Two related facts have been established in the neighborhood demography of racial stratification. One, exposure to disadvantaged residential contexts is strongly associated with a host of quality-of-life indicators. Although disagreements exist on the causal status of neighborhood effects, there is little doubt about the strength or consistency of associations and there is a plausible case for the consequential effects of exposure to neighborhood disadvantage on well-being [1–4]. Second, there is longstanding inequality by race in such residential environments. Black and white households reside in substantially different neighborhoods in the U.S., and the odds of being born into high-poverty neighborhoods is much higher for Black children relative to white children [5]. Taken together, these facts underscore that the neighborhood demography of racial stratification is crucial for understanding inequality in life chances.

However, the traditional focus in neighborhood stratification research is on average residential attainment—the typical residential environments by which individuals are surrounded over extended periods—which we argue obscures important dynamic heterogeneity within and between racial/ethnic groups over time (see also Bader and Warkentien 2016) [6]. Motivated by findings of differential class permeability by race in exposure to disadvantage, the present study goes further by testing for heterogeneity by race in the neighborhood residential trajectories of individuals over time. Building on a body of research on the processes of neighborhood attainment and residential mobility, we view cumulative neighborhood exposure as a sequence over the life course and highlight the differentiated residential trajectories that children follow as they grow up, come of age, and enter young adulthood. We depart from past work in a number of other ways, as well.

First, we focus on dynamic heterogeneity in the residential pathways of exposure to neighborhood poverty among individuals from multiple birth cohorts in Chicago under the influences of different periods of social change. In most neighborhood-focused work, macrolevel influences are largely relegated to the background of empirical focus. However, secular factors (i.e., period contexts) or discrete events specific to a particular period may influence the dynamics of residential moves and set children on different neighborhood trajectories. Our study sheds light on potentially important differences by race between cohorts, drawing on rich, longitudinal data with a multi-cohort design.

Second, our study benefits from a racially and socioeconomically diverse sample in which white, Black, and Latino children are all well represented. Prior research has focused largely on comparing African American and white individuals despite the fact that the Latino share of the population has grown dramatically in recent decades. By following children that lived in Chicago at the start of our study, we also effectively account for city- and state-level characteristics that may vary between places and could influence neighborhood attainment and residential moves, thus allowing us to observe potentially divergent trajectories from children that began in similar urban settings. In particular, our residential history data moves beyond the Black-white binary and allows us to simultaneously examine the neighborhoods of white, Black, and Hispanic children over a sustained period. Note that throughout this paper, we rely on census designations for racial/ethnic groups.

Third, our study period spans nearly three decades almost to the present—our data on residential moves and context begins in the early 1990s and continues through 2018. This nearly three-decade period coincided with a great deal of urban change in Chicago, as well as sociodemographic change on a national level. Since our study treats variation between historical periods as important theoretical terms to be foregrounded in models, this study frame supports our goal of analyzing differential trajectories by birth cohort. Moreover, we are able to follow children through an important period of the life course while also observing how broader structural influences shape residential pathways.

Using these multi-cohort data, we incorporate group-based trajectory methods that allow us to test both the consistency of between-racial group inequality, as well as within-group heterogeneity. Overall, we find remarkable consistency of white advantage and dynamic heterogeneity of nonwhite disadvantage, but these patterns are conditioned by the historical change that differentiates the experience of successive birth cohorts.

## Theoretical motivation

### Neighborhood stratification processes

A large body of research aims to understand processes of neighborhood stratification. One line of inquiry focuses on the residential mobility of people into unequal neighborhood environments by racial and economic status. These studies focus on conditions predicting a residential move, often relying on logistic regression or discrete choice models, where the outcome is a move to specific type of neighborhood. For example, past work examining the movement of individuals between different types of neighborhoods over time has largely focused on the probability of moves, for a single cohort, between poor and nonpoor neighborhoods at discrete points [7–9]. Such studies yield important insights about how individual, family, and extra-household (e.g., neighborhood- or metropolitan area-level) factors interact to induce individual moves [9–14].

Another stream of research focuses on the neighborhood attainment of households which, like the residential mobility literature on choice, examines origin and destination neighborhoods [15–18]. But unlike residential choice models, the neighborhood attainment scholarship focuses more squarely on the characteristics of neighborhoods as outcomes rather than the decision-making process or conditions under which families move—e.g., whether a residential move results in a change in exposure to neighborhood racial/ethnic or socioeconomic composition.

Regardless of approach, research on residential sorting and neighborhood attainment typically describes an average experience, by race or economic status. In this paper, we argue that the focus on average neighborhood attainment obscures potential heterogeneity within- and between racial/ethnic groups in any particular year and over time. There is limited empirical evidence on this issue, but reason to hypothesize that residential trajectories mask considerable differences. For example, two studies use Panel Study of Income Dynamics (PSID) data to examine change in residential environments with a focus on disparities between Black and white individuals. Sharkey (2012) finds that young adults that move out of segregated metropolitan areas experience less economic inequality in their neighborhoods than earlier years, though the trend only lasts through their late 20s [19]. Wagmiller (2013) observes different trajectories between white and Black individuals in their exposure to neighborhood racial composition, with black households experiencing more racially diverse neighborhoods in early adulthood [20]. Both studies suggest that residential environments of Black young adults change over time and that these changes in their residential trajectories may attenuate racial inequality in neighborhood contexts.

There are distinct theoretical reasons supporting our argument on neighborhood trajectories. Although coming from a different tradition, Wilson (1978) theorized about the origins of class stratification, arguing that macrohistorical forces combined to generate heterogeneous groups within Black America. Wilson asserts that while discrimination by race played the primary role in shaping Black Americans’ social lives prior to World War II, the macrohistorical forces in the period thereafter increased the importance of economic factors [21]. Postwar industrial expansion, coinciding with the civil rights movement and newly legislated anti-discrimination laws, made possible economic opportunities previously unavailable to Black households. It is behind this historical backdrop that class stratification among Black Americans began to take shape. A stable Black middle-class emerged as those with greater human capital and formal education were able to take advantage of the economic opportunities during this period, distancing themselves from the spot on the proverbial ladder that their lower-resourced counterparts occupied [21]. The relatively recent development of a non-uniform “Black class structure” that Wilson describes since the postwar period, in which affluent and poor Black individuals are increasingly and meaningfully stratified, carries empirical implications in that it necessitates inquires beyond examinations of a monolithic Black experience [22]. This point is undertaken further in subsequent sociological work demonstrating the differentiated and nuanced experiences of the Black middle class [23–27].

Nevertheless, the intersection of class and race matter; past work also shows that, despite their advances, the rising economic fortunes of the Black middle class does not necessarily dissipate existing disparities with White households. This includes the neighborhoods in which they live, as Black middle-class households are not always able to translate their higher economic status into lower poverty residential environments at the same rate as their White counterparts [25,28,29]. Sharkey (2014) also finds that there has been no change over time in the degree to which majority-Black neighborhoods are surrounded by spatial disadvantage—predominantly Black neighborhoods, regardless of their own socioeconomic composition, continue to be spatially linked with areas of severe disadvantage [30].

Past work attempting to explain how residential outcomes vary between members of different racial/ethnic groups has largely relied on two theories of residential sorting. Place stratification theory, typically used to explain the residential location outcomes of Black households, centers race in explaining why households with higher incomes or socioeconomic states (SES) are unable to actualize their individual economic position into lower-poverty neighborhoods [31]. Place stratification is typically used to explain the residential location outcomes of Black households, but there is some evidence to suggest that there is more heterogeneity in this association between race, class, and residence [32,33]. For example, Freeman (2008) finds that, for Black households, higher socioeconomic status is generally associated with lower poverty residential environments, though their ability to translate higher SES to greater locational attainment changes little between 1970 and 2000 [32]. Spatial assimilation theory, on the other hand, is typically used to explain the residential outcomes of Hispanics and asserts that nonwhite households are able to translate their increasing socioeconomic status into lower-poverty neighborhoods [31]. While this would suggest that class plays a role in differentiating locational outcomes between Hispanic and Black households, there is mixed evidence as to whether this has changed over time [33].

### The racial demography of neighborhood stratification through the lens of the life course

A few recent studies apply a life-course perspective to neighborhood attainment research by asserting that early residential environments are associated with later-life neighborhood contexts. This line of work examines how the sociodemographic contexts of one’s residential neighborhood in childhood [34] and adolescence [35] predict the type of neighborhoods that one attains in later life. Huang et al. (2020) find that children that spend more time in high-poverty neighborhoods are less likely to move from a nonpoor to poor neighborhood and more likely to move from a nonpoor to poor neighborhood [36]. Further, the duration of time spent in poor neighborhoods explains most of the differences in the probability of moves between white and Black adults. Using growth curve models, South et al. (2016) find that neighborhood economic conditions improve in later life more for white, relative to Black, children and that the more economically advantaged adolescent neighborhood environments predicted neighborhood advantages for white individuals in adulthood [35]. The authors of both studies draw on the life course *principle of time and space*, which emphasizes how individual lives are linked to macrolevel events and trends [35,37].

Viewing the racial demography of neighborhood stratification through the lens of the life course is useful for thinking about when and, under what conditions, individuals’ residential environments change or perpetuate [4]. Although the past work described provides important insights about how residential environments unfold as individuals age from childhood to middle age [10,35,36], they do not disaggregate how broader trends and events during different periods might shape residential processes between children that experience life stages in different years [37–40]. Moreover, these studies observe the average residential trajectories [35] or exposures [36] of children over time; we hypothesize that there is more diversity within racial/ethnic groups and between individuals that came of age during different periods.

Experiencing a change in residential environments may result from change at either the individual household or the neighborhood level, or both [5,19,34,41]. Neighborhoods may change in economic status because the incumbent resident population experiences increasing socioeconomic status (SES) or, alternatively, when the in-moving population differs from existing residents along racial/ethnic and SES lines—e.g., during gentrification and neighborhood ascent or as affluent neighborhoods further consolidate their wealth [42–45].

A key underexplored factor, we argue, is that the trajectories of both neighborhoods and individuals, at any given stage, may be influenced by macro-structural conditions tied to that particular period. For example, the disadvantageous impact of the housing crisis of the 2000s (and its fallout) was racially patterned with Black and Hispanic households affected the most by subprime lending practices of that period [46,47]. Hall et al. (2018) find that experiencing a foreclosure during the Great Recession was associated with moving to more disadvantaged neighborhoods, particularly for Hispanics [48]. Such foreclosure-induced migration at the individual level aggregates to reconfigure the sociodemographic makeup of neighborhoods. On a broader secular trend level, other scholars have documented the shifting spatial organization and changing demography of neighborhood poverty since the 1990s, with the proportion of poor neighborhoods in suburbs increasing and, in the case of Chicago, a deconcentration of neighborhood poverty [49–51]. Such period “shocks” and trends likely redirect the residential pathways differently between racial/ethnic groups, and between individuals at different life stages.

Most prior studies also focus on the neighborhood environments of Black and white households [7]. Since the 1990s, however, cities across the U.S. have seen an increasing number of mixed-race and “global neighborhoods” as a greater share of Hispanic and Asian households migrate to urban areas [52–54] under the backdrop of broader sociodemographic change at the local and national level in which white individuals represent a decreasing share of the population and in which income inequality between households and neighborhoods has risen [45,55]. These changes are reshaping neighborhoods and cities, and the question remains as to how these demographic trends affect neighborhood attainment for all racial/ethnic groups, but especially Hispanics. Indeed, some scholars have argued that immigration from Latin America has reshaped the Black-white framework of prior research [56]. We therefore highlight the neighborhood economic trajectories of Hispanic-American individuals compared to African-American and white individuals.

Finally, past work largely captures cumulative exposure to neighborhood poverty over the life course as the proportion of time that individuals spend in poor and nonpoor neighborhoods or the average level of neighborhood poverty of some extended period of time (often childhood) [8,14,36]. While capturing a temporal dimension largely overlooked in early neighborhood research (i.e., duration of exposure) [4], such summary measures may mask dynamic heterogeneity in the sequence and timing of exposure to residential environments between subgroups. Timberlake (2007) uses period life tables to predict transitions into neighborhood poverty during childhood, showing important change over time in Black and white children’s predicted exposure to neighborhood poverty (or affluence) at different stages of childhood and between periods [57]. This study relies on data prior to 1997, however, after which broader sociodemographic change has occurred, and it focuses on the individual’s predicted duration of time in neighborhood poverty rather than focusing on identifying the trajectories of neighborhood poverty themselves.

Advancing prior work, the present study tests for potential differences in neighborhood trajectories between historical periods and by race, using data that spans from the early 1990s to 2018. To do so, we examine the overall sequence of neighborhood exposure to poverty that individuals from different birth cohorts and racial/ethnic groups experience across stages of the adolescent and early adult life course.

## Data, analysis plan, and measures

### Data

To answer our questions, we draw on data from the Project on Human Development in Chicago Neighborhoods (PHDCN), a representative longitudinal cohort study of 6,207 children and their caregivers that began in the 1995. Our study was approved by the Harvard University Committee on the Use of Human Subjects in Research.

PHDCN employed a two-stage sampling procedure to first identify 343 neighborhood clusters (NCs) in the city of Chicago, then draw a random, stratified sample of 80 NCs, and second, sampling within these 80 NCs from randomly selected and screened households for children in seven age cohorts (zero [birth], three, six, nine, twelve, fifteen, and eighteen). The resulting cohort populations from initial collection were representative of the diversity of children in Chicago in 1995 [58]. This multi-cohort design, which includes children born up to 17 years apart, and stratified by neighborhood socioeconomic status and race/ethnic composition, makes the PHDCN an ideal data source for analyzing intra- and inter-cohort differentiation in individual’s neighborhood trajectories [59]. See Sampson et al. (2022) for further details about the design, implementation, and unique features of the PHDCN’s longitudinal multi-cohort design [58].

Three waves of extensive in-home interviews and assessments were collected over a span of seven years—wave 1 in 1995–1997, wave 2 in 1997–1999, and wave 3 in 1999–2002—with relatively high retention (approximately 75%) as children were followed, regardless of whether they remained in Chicago. These interviews generated important information about the location of current and past residences (including locations for inter-wave years), which could be used to construct annual residential histories for each child over time.

In 2012 and early 2013, the Mixed-Income Project (MIP) randomly sampled from the original birth (0 y/o) and 9- to 15-year-old cohorts that were last interviewed in wave 3, then relocated and re-interviewed sampled children via in-person (~60 percent), phone, and electronic interviews. For ease, we refer to this follow-up with a subset of baseline respondents as PHDCN wave 4. During wave 4 collection efforts, investigators collected additional information about respondents’ current residence, as well as previous residential moves in the preceding years. The follow-up efforts resulted in a 63 percent response rate of eligible cases and similar racial/ethnic composition of PHDCN at baseline (roughly 19 percent white; 37 percent Black; 40 percent Hispanic; 4 percent other race). The birth cohort comprised nearly 36 percent of the wave 4 sample (n = 378), while “older” respondents were relatively evenly distributed among the 9- (n = 226), 12- (n = 236), and 15-year-old (n = 217) cohorts. These four groups in wave 4 were selected, in part, to enable cross-cohort analysis between respondents that varied in their life course timing and experiences.

Location efforts resumed in 2018 and 2019. NORC at the University of Chicago attempted to follow up with all PHDCN respondents that were located during wave 4 and were able to gather detailed information on respondents’ current residential address (in 2018/19) for about 83 percent of respondents. We appended this information to the residential history data that we constructed for wave 4 respondents from 1995 to 2013. Additionally, for respondents in the 9-, 12-, and 15-year-old cohorts, we were able to retroactively gather residential data for 4–5 years prior to baseline, essentially providing a file with annual residential location information from as early as 1990 to as late as 2019. After geocoding respondents’ addresses in each year, we then joined them to tract-level decennial census and American Community Survey (ACS) data, interpolating values during intercensal years prior to the introduction of the ACS in 2005. The resulting file contained roughly three decades of information, for each cohort, on when and where respondents moved, and on the sociodemographic contexts of their residential neighborhoods in each year (N = 1057). Respondents ages ranged from 20 to 37-years-old during the final year of data collection (see Table 1). Our cohorts in our file closely mirrored distribution from wave 1 in 1995 (33 vs. 35 for the 0-year-old birth cohort; 67 vs. 65 for the combined 9-, 12-, 15-year-old birth cohort).

**Table 1:** Overview of analysis file by cohort.

		Younger Cohort	Older Cohort		
		0	9	12	15
		*32*.*5%*	*21*.*0%*	*25*.*3%*	*21*.*2%*
*Year*		* *	* *	* *	* *
	at Age 5	1999–2001	1990–92	1987–89	1984–86
	at Age 9	2003–05	1994–96	1991–93	1988–90
	at Age 23	2017–19	2008–10	2005–07	2002–04
*Age*					
	in 1995	0–1*	8–10	11–13	14–16
	in 1998	2–4	11–13	14–16	17–19
	in 2001	5–7	14–16	17–19	20–22
	in 2008	12–14	21–23	24–26	26–28
	in 2011	15–17	24–26	27–29	30–32
	in 2013	17–19	26–28	29–31	32–34
	in 2018	22–24	32–34	35–37	38–40
*Age Range*, *1995–2018*					
		0–1 to 22–24 y/o	8–10 to 32–24 y/o	11–13 to 35–37 y/o	14–16 to 38–40 y/o
*Race (percent)*					
	White	36.0	19.9	24.8	19.2
	Black	31.5	20.3	29.3	19.0
	Hispanic	30.6	23.1	21.3	24.9
	Other	42.4	12.1	30.3	15.2

*Note*: Residential information for pre-baseline (1995) years were collected from in-depth interviews with primary caregivers and/or sample members. Sample percentages refer to the analysis sample (N = 838).

*Roughly 96 percent of the birth cohort (0) was born in 1995 and 1996 (59 and 37 percent, respectively), but a small percentage was born in 1994 (~4 percent).

### Analysis plan

Our analysis proceeds in several stages with each part building sequentially on previous steps. We first aim to understand the average sociodemographic contexts of children’s residential neighborhoods, and whether they vary by race and period cohort. We analyze descriptive trends in sample members exposure to neighborhood poverty by race and cohort by year, from 1995 to 2018. For these analyses, we combine the 9-, 12-, and 15-year-olds into a single cohort (“older” cohort) that we compare to the birth cohort (“younger” cohort) so that we can make meaningful inter-cohort inferences about children that experience stages of the life course in substantively different periods. Results from these unadjusted analyses indicate potential within- and between-group differences in residential trajectories, motivating our subsequent analyses. From there, we place our question in a multivariable framework.

### Model: Group-based trajectory analysis

Past work on this topic often takes analytic approaches such as period life tables, logistic regression, or transition matrices [8,10,57]. Often, these studies employ growth curve models (South et al. 2016), which are effective for empirically describing individual-level variability in trajectories of exposure to high-poverty neighborhoods over time, but are limited in that they allow the residuals to vary around the estimate for a single trajectory. By estimating mean trends, these methods assume that all individuals in the population follow a similar functional form in their residential pathways—i.e., one trajectory shape that is assumed to “fit all” (Nagin and Odgers 2013:115)—thereby potentially overlooking important within-group differences [60].

Our theoretical motivation centers on testing whether this heterogeneity exists in the residential pathways themselves that children from different birth cohorts experience, which requires a different methodological approach than used in prior work in this area, namely one that is *group*-based and that does not carry these stated assumptions. Thus, to answer fundamentally different questions about uncovering potential heterogeneity in neighborhood poverty trajectories among all sample members, we rely on group-based trajectory models (GBTM), a method that allows for errors to cluster around several trajectories. This technique is particularly useful for identifying unexpected or latent trajectories and provides a more refined classification of exposure based on temporal order [6]. GBTM offers a meaningful alternative to prior work in that these models classify individuals into different residential trajectories based on similarity in their actual propensity to live over time in high-poverty neighborhoods.

GBTM is a specialized application of finite mixture modeling that categorizes clusters of individuals that experience similar trajectories over time into classes, identifying statistically similar patterns of residential trajectories by subgroups [61]. For our analysis, we perform group-based trajectory prevalence models (also called “risk” models) to identify high-poverty neighborhood pathways among individuals over a 2.5-decade period (1995–2018), focusing on the pathways that emerge from childhood to young adulthood (i.e., ages 9 to 27). We performed our group-based trajectory modeling in Stata (version 16) using the *traj* command.

Essentially, GBTM uses maximum likelihood estimation to jointly estimate the shape of trajectories and the proportion of the sample in each trajectory. Each respondent is assigned a probability of belonging to each group. GBTM fit statistics help guide researchers to the optimal number of groups by showing if the addition of one more trajectory improves the fit of the model.

GBTM can fit any number of groups and functional forms. Following Nagin (2005), we specified the final number of groups and functional form based on model fit (BIC) and group size (smallest group >5 percent of full analysis sample) after empirically testing 1 to 7 groups with various functional forms (linear, quadratic, cubic, and combinations of the three forms), as well as the interpretability of the model for explaining the data [62]. All models had an average predicted probability of group membership (APP) greater than 0.9, which was well above the recommended threshold (> = 0.7) for best-fit model consideration [62]. Appendix Figure S1 in S1 Appendix further illustrates this point, displaying kernel density plots of the predicted probability of membership into the observed trajectory for all children included in group-based trajectory prevalence models for residence in high-poverty neighborhoods from 1995 to 2018, demonstrating that our GBTM model predicts membership well for both cohorts and for white, Black, and Hispanic sample members. Importantly, Nagin and Odgers (2013) suggest that researchers pair these fit statistics with their theoretical understanding of the process under focus [60]. Our model selections represent the most parsimonious summary of the data after accounting for these recommendations. Guided by these recommendations, we selected 5-group models for neighborhood poverty trajectories, relying on cubic age terms. We emphasize Nagin and Odgers’s (2013:118) view that “trajectory groups are just approximations of a more complex reality” and that researchers should “move away from interpretations of trajectory groups as literally distinct entities” [60]. See Appendix Table S1 in S1 Appendix for details on diagnostics and model selection.

Our group-based prevalence analysis relies on several measures to identify trajectories of neighborhood poverty. Using decennial census and 5-year ACS data, we construct sociodemographic measures of neighborhood context, which we capture annually from 1991 to 2013, and again in 2018. We perform a prevalence GBT model, which uses the logit link function and relies on a binary outcome for neighborhood poverty for each sample member and at each time point (in our analysis, we draw on data from 1991 to 2018, but use an individual’s *age* as our time variable). To do this, we construct a dichotomous measure of *high-poverty* neighborhoods denoting whether the poverty rate of a sample member’s residential neighborhood is above 20 percent, following past work [36,57].

In our GBT models, we further account for several time-invariant individual measures and baseline family characteristics captured at wave 1 (in 1995), drawing on the PHDCN. We include three individual time-invariant binary measures: *race*, *cohort membership*, and *gender*. Race of the child is reported by the primary caregiver during the initial interview and consists of four categories: White, Black, Hispanic, and other race. As described above, for our dichotomized measure of birth cohort membership, we classified those in cohort 0 (infancy) as members of the younger cohort (= 0) and those in the 9-, 12-, and 15-year-old birth cohorts as the older cohort (= 1). *Gender* categorizes a child as female or male, as reported by the primary caregiver in wave 1. We further include baseline family factors (drawn from PHDCN) known to be related to later economic attainment, such as binary indicators for *homeownership*, whether the primary caregiver (PCG) earned a *college degree*, *employment status* of the household head (employed or not employed), and *family size* (whether a household has five or more members). We also construct a dichotomous baseline measure denoting whether the child’s family is high-income. Family income designations are drawn from PHDCN using a 7-category household income measure observed at baseline (1995). The top category captures families with incomes greater than 50,000 in 1995, which we define as *high-income*. Based on the distribution of the baseline family income variable, we categorize sample members in the highest income category (>50,000 or more) as “high income.” We further include measures capturing exposure to violence and family personal and institutional troubles. We used factor analysis to calculate a baseline *family composite score*, which summarizes answers to six correlated interview questions from the PHCDN about family personal and institutional troubles—number of family members currently in jail or prison (0–3); that have had trouble with the law (0–7); that have had trouble with their job, fights or school (0–6); treated for drug use or emotional problems (0–6); that have alcohol use (0–6) or drug use (0–7) that led to trouble with family, jobs, health, or the law—into a single continuous measure, with higher values indicating greater troubles (mean = 0, sd = 1). *Exposure to violence* is a dichotomous measure indicating exposure at baseline. For further details on measurement see Neil and Sampson (2021, Table 1) [59].

### Model: Multinomial logistic regression

Finally, after reducing the heterogeneity in the individual-level neighborhood poverty trajectories to a set of distinct group trajectories, we perform multinomial logistic regression models to analyze how membership in the different trajectory groups varies as a function of time-invariant and baseline predictors measured prior to the start of the trajectories (Nagin 2005). For our multinomial logistic regression, we construct a categorical measure denoting the five neighborhood poverty trajectory groups (identified via GBTM) as our outcome variable. In these models, we include all time-invariant and baseline measures from GBTM as predictors (described above). Additionally, we account for whether a child was residing in a high-poverty neighborhood at baseline (0 = not high poverty; 1 = high-poverty).

All coefficients from multinomial logistic regression models are reported as log-odds. This approach allows us to simultaneously estimate both the relationship between baseline time-invariant covariates with the probability of trajectory group membership and the parameters of the trajectories themselves [62]. This enables us to examine whether key childhood factors prior to the start of the observation period for the trajectory analysis are associated with the probability of membership in each residential trajectory group. Note that while the information derived from these time-invariant covariates shows which of the neighborhood trajectories a child is most likely to follow, it does not define the specific form of that trajectory over time.

### Analysis sample characteristics

Table 1 presents an overview of our sample, displaying the age of each cohort member in select years. Our baseline year is 1995, though we collect data on residential history beginning in 1990 (for older birth-cohort members). We are thus able to register neighborhood context for all but the oldest sample members during their childhood years and can track the youngest sample members through their mid-20s.

### Missing data

Similar to other longitudinal modeling techniques, group-based trajectory models retain respondents even if they are missing from some waves, so missing data at a given age should not affect trajectory development [62,63]—i.e., an individual remains in the sample even with a single wave of non-missing data on the outcome of interest. In our analysis, the sample member with the most non-missing neighborhood poverty data contributed data points for four time points. (Recall that the GBT model uses age of the individual as its time variable and only observes respondents when they are between the ages of 9 and 27). Roughly 97 percent of respondents had neighborhood poverty data for at least nine time points and the median percentage of missing neighborhood poverty for respondents was zero.

On the other hand, missing covariate data that is not random may introduce bias in multinomial regression models that analyze the association of these risk factors with the identified trajectory groups. For our analysis, we eliminated sample members that had missing data on the baseline family characteristics included in our models. This restriction reduced the full geocoded sample (N = 1057) to 838 sample members. Appendix Table S2 in S1 Appendix compares the characteristics of the full geocoded sample to our final analysis sample and shows that the two samples have very similar racial composition (overall and by cohort). The analysis sample has a slightly higher proportion of children from the youngest birth cohort, but otherwise has a comparable distribution of birth cohorts as the full sample. Additionally, sample means for all measures included in our GBTM and multinomial regression models are strongly aligned between the analysis and full geocoded files which alleviate concerns about potential bias (see Appendix Table S2 in S1 Appendix).

## Findings

### Descriptive analyses: Unadjusted mean poverty trajectories over time, 1995–2018

**Fig 1:**
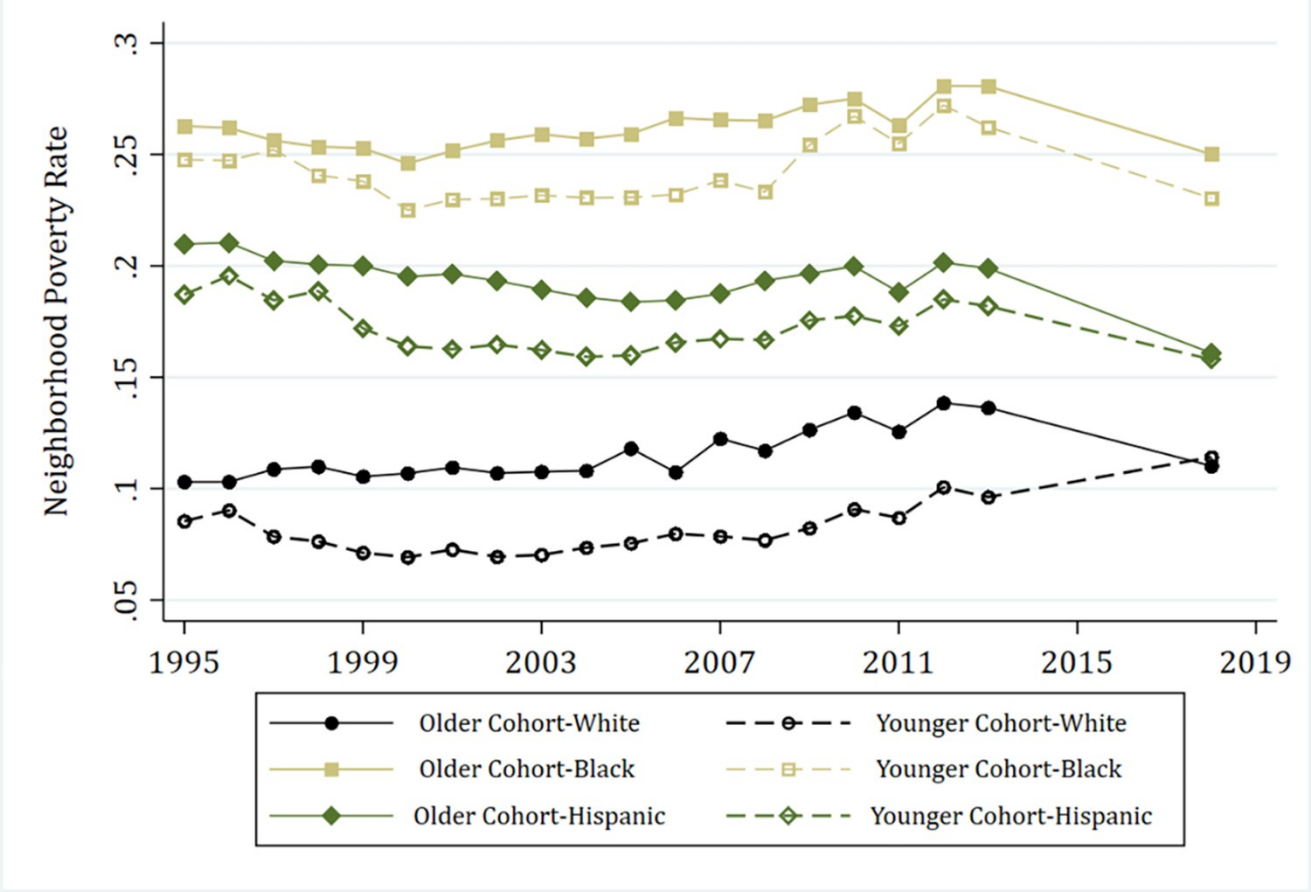
Mean neighborhood poverty rate by cohort and race, 1995–2018. *Note*: Proportion poverty denotes the share of poor residents that live in a census tract (measured in each year). Tract-level neighborhood data come from decennial census in 1990 and 2000, and American Community Survey (ACS) 5-year estimates from 2005–09 to 2014–18. We use the midpoint of ACS estimates for year (i.e., we use ACS 2005–09 for the year 2007) except for ACS 2014–18, which uses the endpoint as year (i.e., 2018).

We begin by descriptively analyzing trends in neighborhood sociodemographic contexts among sample members by race and period cohort from 1995, the baseline of the PHDCN, to 2018. Fig 1 plots the average neighborhood poverty rate, by race, for the *younger* (0-year-old [birth] cohort) and *older* (9/12/15-year-old) cohorts. As expected, we observe differences in mean neighborhood poverty race between white, Black, and Hispanic children at baseline that continues over the course of the study period. We also observe differences within racial/ethnic groups between cohorts. The disparity in exposure to neighborhood poverty rate between cohorts widens beginning in the mid-1990s and continues until around 2008, coinciding with the period of the Great Recession and its aftermath. After 2010, the period cohort trend lines for white and Hispanic individuals converge, while they begin widening again for Black individuals.

### Group-based trajectory models

Does childhood neighborhood context follow people from childhood through young adulthood? Our unadjusted descriptive analyses provide suggestive evidence of meaningful relationships between race and cohort on children’s exposure to neighborhood poverty in each year and over time. Black and Hispanic sample members seem to experience more heterogeneity in their trajectories of neighborhood exposure to poverty.

Our next set of analyses relies on group-based trajectory models (GBTM) to identify potential latent subgroups experiencing similar trajectories of exposure to residential contexts during the early life course. It could be the case that there are other unifying characteristics aside from race and cohort that group sample members into similar trajectory classes.

**Fig 2:**
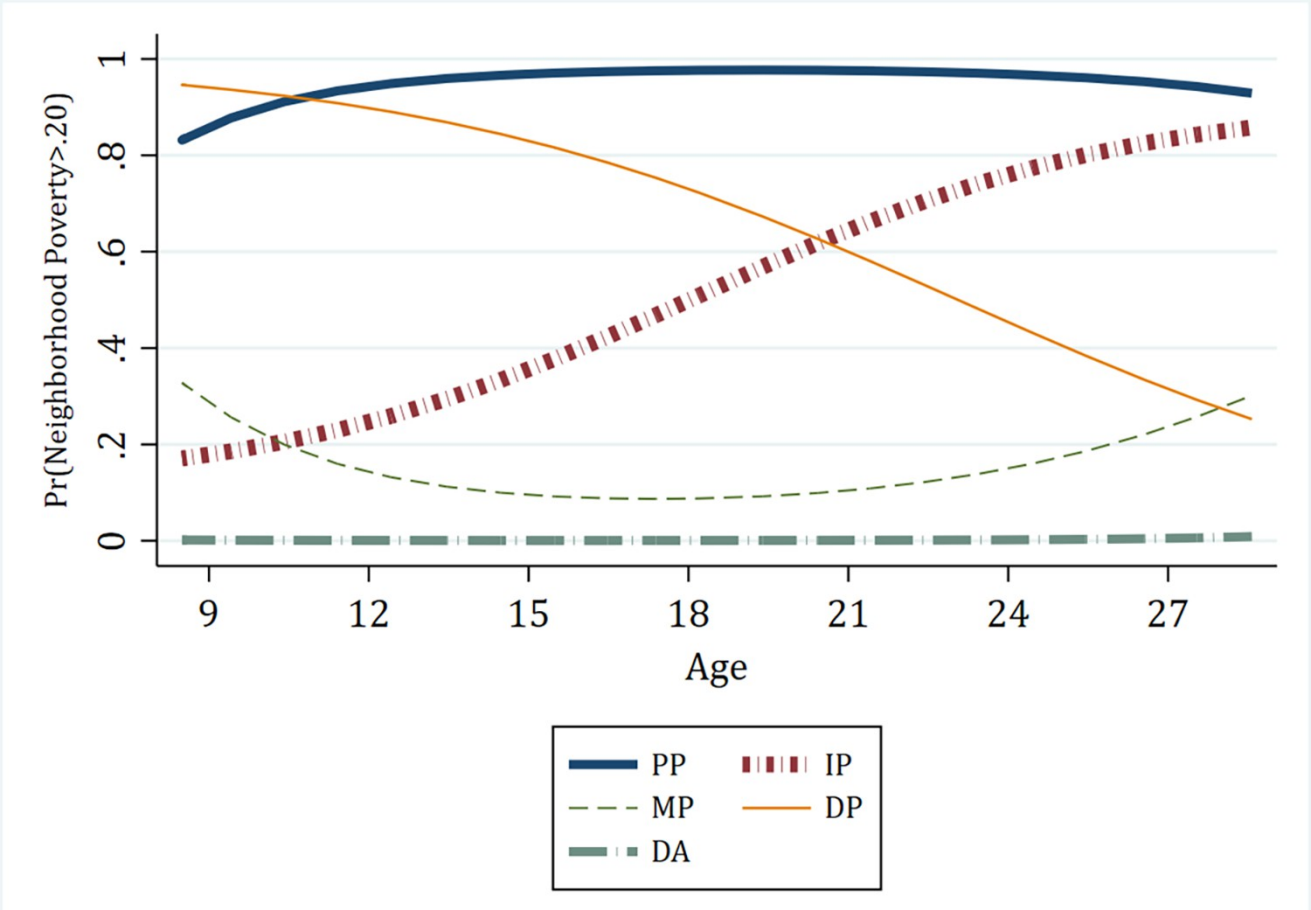
Residential trajectories in the risk of exposure to high-poverty neighborhoods. *Note*: 1) PP = persistent poverty (18.5%); 2) IP = increasing poverty (18.7%); 3) MP = marginal poverty (17.1%); 4) DP = decreasing poverty (18.9%); 5) DA = durably advantaged (26.8%). Observed residential trajectories drawn from a group-based trajectory model (cubic age term) predicting the risk of exposure to high-poverty (>.20) neighborhoods, by age. Percentages in legend correspond to the posterior probability of each trajectory. This is calculated by aggregating each person-year observation’s predicted probability of membership in each trajectory group.

To illustrate findings, we generated figures that display the trajectory subgroups identified in our GBT models using the *trajplot* command in Stata (version 16). Fig 2 shows results from a prevalence GBTM analysis identifying trajectories in the risk of exposure to high-poverty (> 20 percent) residential contexts from childhood through young adulthood (age 9 to 27). Our models rely on an analysis sample that includes the oldest cohort members (15-y/o birth cohort) for which we have less information during early childhood years, thereby taking advantage of GBTM’s ability to use all of the information to draw out distinct subgroups in the data. To ensure our results were not driven by noise in our data, we performed as a robustness check models excluding the 15-year-old members of the older cohort, which produced the same trajectories. Because the final wave of data (2018) contained a substantially higher share of missing data than previous years, we also performed models that used 2013 as the end year. Trajectories mirrored those observed when using the sample that included all years. Note also that we report results based on unweighted data because they are more efficient than the corresponding weighted estimates and our main quantities of interest are the longitudinal trajectories within our sample. We also condition on race and baseline economic status, two key factors related to the original design stratification. As a check, however, we plotted the mean neighborhood poverty level across all study years using the weighted sample, then compared results to those drawn from our analysis sample (Appendix Figure S2 in S1 Appendix).

We identified five poverty trajectories. Two poverty trajectories are characterized by stability while three feature changing exposure to neighborhood high-poverty contexts at different stages of the early life course. Children in the largest group, the *durably advantaged* (DA) trajectory (27.6 percent), experience effectively zero risk of residing in a neighborhood with greater than 20 percent poverty as they age through their 20s. Conversely, the *persistent poverty* (PP) trajectory is characterized by increasing propensity of exposure to high-poverty neighborhoods in late childhood followed by continuous, severely elevated propensity of residence in high-poverty neighborhoods. Three trajectories are characterized by changing propensity of high-poverty neighborhood exposure over the life course. For children in the *increasing poverty* (IP) trajectory, the low propensity of residing in high-poverty contexts increases sharply from adolescence to adulthood. On the other hand, those in *decreasing poverty* (DP) trajectories experience the opposite trend as their substantially elevated risk of high-poverty neighborhood exposure decreases dramatically beginning in adolescence. The probability of exposure fluctuates moderately during late childhood and early adulthood for individuals in the *marginal poverty* (MP) trajectory, though the probability remains lower at nearly all ages compared to the IP and DP trajectories.

To alleviate concerns that our group-based prevalence models were sensitive to our threshold definition for high-poverty neighborhoods (>.20), we also plotted the actual neighborhood poverty rate for each of the classes identified via GBTM (Appendix Figure S3 in S1 Appendix). The shape of the trajectories mirrored what we found in our group-based prevalence models, which gave us confidence that our results were not sensitive to individuals hovering just above or below the neighborhood high-poverty threshold.

### Descriptive characteristics of high-poverty trajectory groups

Extending our GBTM analysis, Table 2 displays baseline descriptive characteristics of the five high-poverty residential trajectories. Sample members in the DA trajectory are disproportionately white and the most advantaged with respect to exposure to high-SES, low-poverty neighborhood contexts, an advantage that persists from childhood through young adulthood. Conversely, the PP trajectory is predominantly comprised of Black, lower-SES, and older cohort sample members. A majority of children’s parents had a college degree at baseline for all trajectory groups, though the proportion of parents with a college degree is highest for the DA group. Note that baseline family SES is similar for IP and DP trajectories, with the proportion of high-income in the IP group slightly higher than DP. As with PP trajectories, a moderately larger share of Black sample members (relative to their overall representation) is grouped in IP trajectories. Most Hispanic sample members are classified in the MP trajectory. While the risk of high-poverty neighborhood exposure is lower for MP groups relative to IP and DP, so, too, is the average baseline parental income and proportion of homeowners (though the share of MP households with a college degree is slightly higher).

**Table 2:** Descriptive statistics of residential (high-poverty) trajectories by race and cohort.

		PP		IP		MP		DP		DA		Overall	
		mean	*sd*	mean	*sd*	mean	*sd*	mean	*sd*	mean	*sd*	mean	*sd*
*Gender distribution*													
	Male	0.47	*0*.*50*	0.50	*0*.*50*	0.49	*0*.*50*	0.41	*0*.*49*	0.55	*0*.*50*	0.49	*0*.*50*
	Female	0.53	*0*.*50*	0.50	*0*.*50*	0.51	*0*.*50*	0.59	*0*.*49*	0.45	*0*.*50*	0.51	*0*.*50*
*Race/Ethnicity*													
	White	0.03	*0*.*16*	0.11	*0*.*32*	0.13	*0*.*34*	0.05	*0*.*22*	0.49	*0*.*50*	0.19	*0*.*39*
	Black	0.65	*0*.*48*	0.49	*0*.*50*	0.30	*0*.*46*	0.42	*0*.*50*	0.11	*0*.*31*	0.37	*0*.*48*
	Hispanic	0.31	*0*.*47*	0.35	*0*.*48*	0.57	*0*.*50*	0.49	*0*.*50*	0.32	*0*.*47*	0.40	*0*.*49*
	Other	0.01	*0*.*08*	0.05	*0*.*22*	0.00	*0*.*00*	0.03	*0*.*18*	0.08	*0*.*28*	0.04	*0*.*19*
*Birth Cohort*													
	Younger	0.12	*0*.*32*	0.45	*0*.*50*	0.20	*0*.*40*	0.34	*0*.*47*	0.44	*0*.*50*	0.32	*0*.*47*
	Older	0.88	*0*.*32*	0.55	*0*.*50*	0.80	*0*.*40*	0.66	*0*.*47*	0.56	*0*.*50*	0.68	*0*.*47*
*Baseline family factors*													
	Owns home	0.33	*0*.*47*	0.42	*0*.*49*	0.46	*0*.*50*	0.34	*0*.*47*	0.69	*0*.*46*	0.47	*0*.*50*
	Employed	0.58	*0*.*50*	0.67	*0*.*47*	0.63	*0*.*49*	0.69	*0*.*46*	0.75	*0*.*43*	0.67	*0*.*47*
	High-income	0.10	*0*.*31*	0.23	*0*.*42*	0.21	*0*.*41*	0.13	*0*.*33*	0.56	*0*.*50*	0.28	*0*.*45*
	Parent BA	0.04	*0*.*19*	0.08	*0*.*27*	0.12	*0*.*33*	0.08	*0*.*27*	0.35	*0*.*48*	0.15	*0*.*36*
	Composite family score	0.08	*1*.*19*	-0.04	*0*.*88*	0.14	*1*.*23*	-0.09	*0*.*84*	-0.04	*0*.*85*	0.00	*0*.*99*
*Neighborhood factors (in 1995)*													
	Neighborhood % poverty	30.76	*12*.*93*	17.76	*9*.*69*	19.06	*8*.*79*	29.91	*11*.*25*	9.16	*5*.*49*	20.27	*12*.*88*
	Neighborhood % White	11.66	*14*.*74*	23.67	*24*.*27*	27.08	*26*.*31*	16.91	*17*.*82*	56.10	*25*.*96*	29.70	*28*.*26*
	N	153		159		137		158		231		838	* *

*Note*: High-poverty (>20 percent) neighborhood trajectories drawn from group-based trajectory (logit) models using all sample members with sufficient data across waves (N = 838). We assigned sample members into the trajectory group with their highest predicted probability of membership. Most predicted probabilities were greater than .90 (see Appendix Figure S1 in S1 Appendix). All baseline family factors were observed at wave 1 of the PCHDN (1995). We used factor analysis to calculate the baseline *family composite score*, which summarizes answers to six correlated interview questions about family trouble into a single measure with higher values indicating greater family exposure to drug and alcohol use, punitive systems, and legal trouble (mean = 0, *sd* = 1). *High-income* is a dichotomous indicator denoting household income greater than 50,000. *Family size* is a binary measure denoting a household size with five or more members. *Younger cohort* was born between 1994 and 1996; *older cohort* consists of children that were generally 9, 12, and 15 from 1994–96 (see Table 1).

DA = durably advantaged; PP = persistent poverty; IP = increasing poverty; MP = marginal poverty; DP = decreasing poverty.

**Fig 3:**
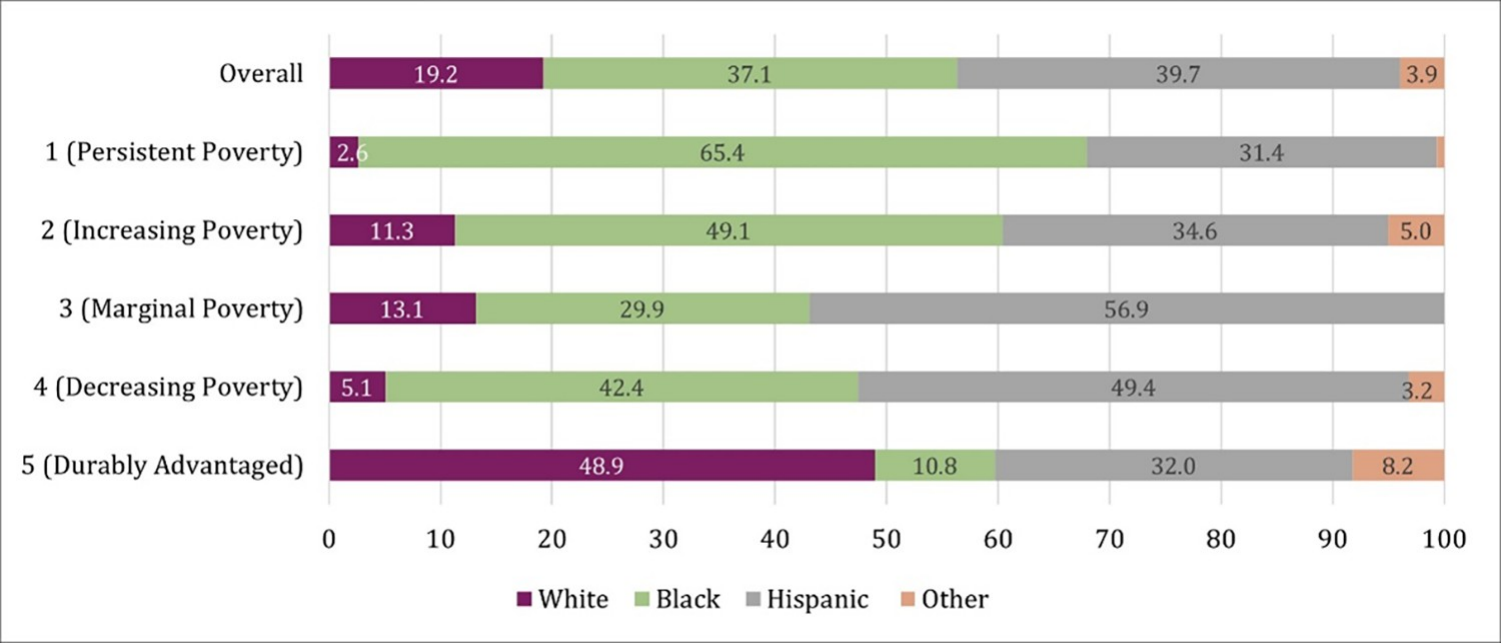
Observed vs. expected racial composition in high-poverty trajectory groups. *Note*: *Overall* refers to the racial composition of the analytic sample used for group-based trajectory models. If sample members were evenly distributed by race into the trajectory groups, we would expect the distribution for each of the five trajectory types to mirror the *Overall* distribution.

**Fig 4:**
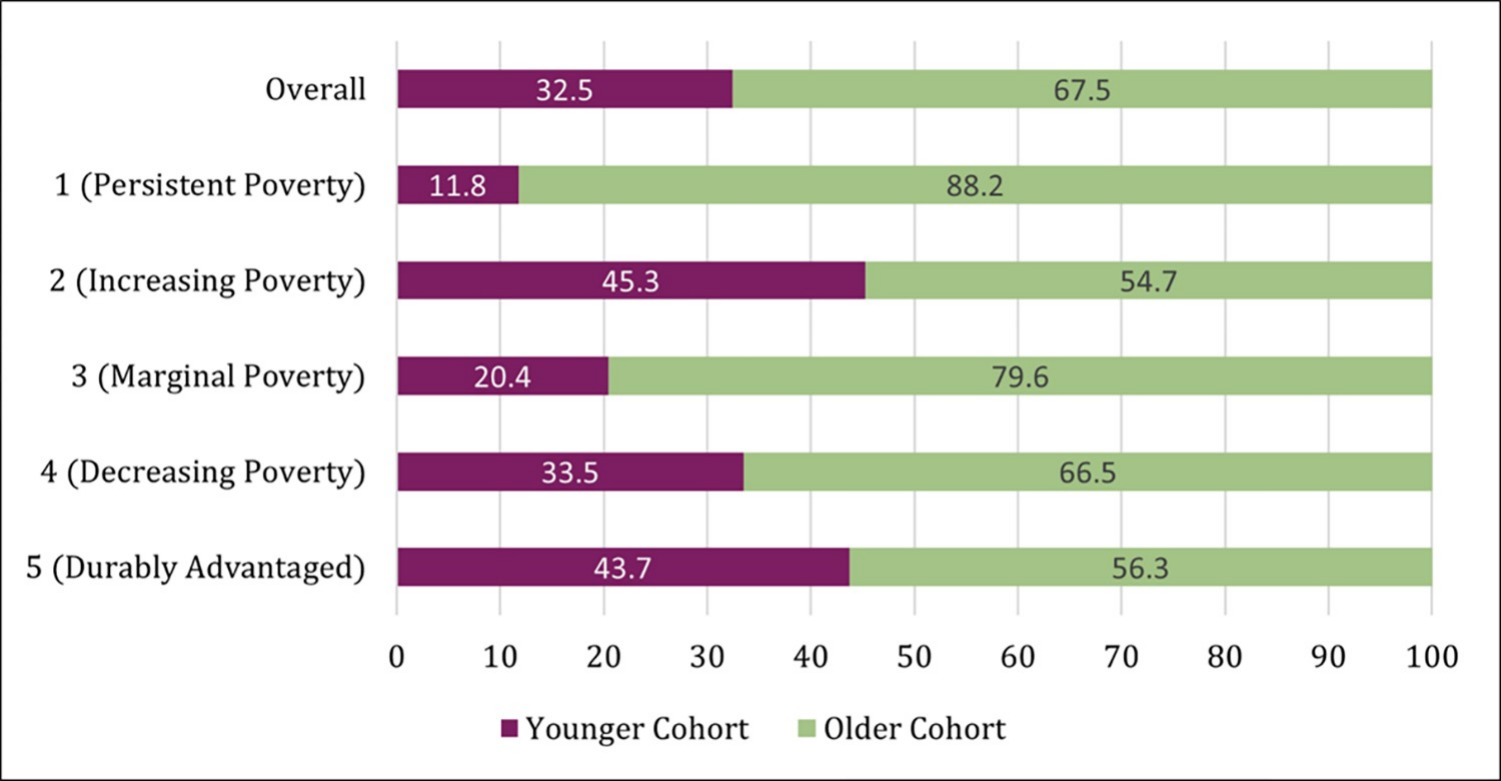
Equally distributed vs. observed cohort composition in high-poverty trajectory groups. *Note*: *Overall* refers to the cohort (period) composition of the analytic sample used for group-based trajectory models. If sample members were evenly distributed by cohort into the trajectory groups, we would expect the distribution for each of the five trajectory types to mirror the *Overall* distribution.

Drawing on descriptive statistics presented in Table 2, Figs 3 and 4 illustrate the disparities in how groups are represented in high-poverty residential trajectories.

Fig 3 displays the extent to which white sample members concentrate into advantaged trajectory groups relative to what we would expect if all racial/ethnic groups were distributed equally, given their representation in our sample. In terms of racial/ethnic composition in high-poverty neighborhoods, although white sample members represent just over 19 percent of the analytic sample, they comprise nearly half of the durably advantaged trajectory type (48.9 percent). This is most evident when we display these findings as observed-expected ratios (see Appendix Figure S4 in S1 Appendix). White sample members are represented in the durably advantaged class at a rate of 2.5 times more than we would expect if racial/ethnic groups were distributed into each class proportional to their overall representation in the sample. On the other hand, we observe that Black sample members are overwhelmingly overrepresented in the persistent poverty trajectory relative to what we would expect if all groups were distributed in equal measure (65.4 to 37.1). Notably, however, Black sample members are also slightly overrepresented in poverty trajectories experiencing both increasing (IP) and decreasing poverty exposure (DP). This suggests more dynamic permeability and heterogeneity in the trajectories of Black sample members relative to the trajectories of white sample members.

Interestingly, Hispanic sample members are overrepresented in the trajectory group characterized by marginal poverty—an overall low probability of high-poverty neighborhood residence, but with modestly increasing probability during the transition to young adulthood (MP). Hispanic sample members are even more clustered in the group experiencing decreasing poverty exposure (DP). The changing fortunes of Hispanic sample members could be the result of intergenerational differences within families as they migrate into lower-poverty contexts after leaving their childhood and adolescent neighborhoods. It could also indicate important differences from unobserved macrolevel influences between Hispanic respondents that experienced the transition to adulthood during different periods. Finally, it could also be the case that higher-SES residents are moving into Hispanic sample members’ existing neighborhoods, thus changing the poverty contexts while sample members age in place. Gentrification is an example of this type of residential sorting process, and this explanation is consistent with prior research on gentrification of Chicago’s Hispanic neighborhoods since the 1990s (Hwang and Sampson 2014). This is also consistent with earlier descriptive findings (Fig 1) which provided suggestive evidence of cohort differences between younger and older Hispanics in their mean neighborhood poverty contexts since 1995.

The striking imbalance in white sample members’ representation in the trajectory groups (they are substantively absent from the three residential pathways characterized by higher propensities to reside in high-poverty neighborhoods) underscores white children’s seemingly intractable advantage–their privileged starting point generally follows them through the early life course.

Fig 4 displays observed and expected cohort membership into high-poverty trajectory groups. (Alternatively, Appendix Figure S5 in S1 Appendix displays these results as observed-expected cohort composition ratios.) Here, we observe both between-cohort differences in predicted group membership and within-cohort differences in the shape and direction of high-poverty trajectories. While older cohort members represent 67.5 percent of the GBTM sample, they make up over 88 percent of the PP group and nearly 80 percent of the MP group. Just under a third of sample members were born into the younger cohort, but roughly 4 out of 9 experience IP and DA trajectories.

Summarizing our key findings, Black children have the highest amount of within-race variation. Hispanic children have some variation but differ from Black and white children in that they are far more likely to be in the “middle” trajectory. White children have little internal variation and are overwhelmingly durably advantaged with respect to their residential environments. Children coming of age more recently (younger cohort) tend to experience more advantaged neighborhood trajectories relative to those born earlier (older cohort).

### Multinomial logit regression predicting trajectory groups

Finally, we further explore descriptive characteristics of trajectory groups, but from a multivariate framework. Table 3 displays results from multinomial logistic regression models (MNL) that simultaneously predict membership in high-poverty neighborhood trajectories based on time-invariant covariates and baseline family characteristics, as well as the parameters of the five group trajectories [62]. This analytic approach allows us to examine which factors in early childhood are associated with the residential pathways that individuals will follow through the early life course. The coefficients are reported as log odds, with the durable advantage (DA) trajectory, characterized by persistently low propensity of exposure to high-poverty neighborhood contexts, as the reference group.

**Table 3:** Multinomial logistic regression of risk factors on high-poverty neighborhood trajectories (baseline outcome = durably advantaged (DA))^a^.

		PP		IP		MP		DP	
		Log odds	[95% CI]	Log odds	[95% CI]	Log odds	[95% CI]	Log odds	[95% CI]
*Ref*. *category*: *DA*									
Individual time-invariant factors									
	White	(ref)		(ref)		(ref)		(ref)	
	Black	3.95***	[2.70, 5.20]	2.85***	[2.08, 3.63]	2.21***	[1.40, 3.02]	2.60***	[1.55, 3.66]
	Hispanic	1.34*	[0.13, 2.54]	0.86*	[0.19, 1.54]	1.24***	[0.56, 1.91]	1.01*	[0.04, 1.98]
	Older (9/12/15) Cohort	(ref)		(ref)		(ref)		(ref)	
	Younger (0) Cohort	-2.02***	[-2.76, -1.28]	0.07	[-0.46, 0.60]	-1.17***	[-1.76, -0.57]	-0.57+	[-1.20, 0.06]
	Female	(ref)		(ref)		(ref)		(ref)	
	Male	0.1	[-0.50, 0.69]	0.09	[-0.41, 0.60]	0.05	[-0.47, 0.56]	-0.37	[-0.95, 0.21]
Baseline measures									
	Composite family score	0.04	[-0.27, 0.35]	0.03	[-0.25, 0.31]	0.22	[-0.04, 0.47]	-0.13	[-0.46, 0.20]
	Parent college degree (BA)	-1.59**	-2.71, -0.48]	-1.71***	[-2.52, -0.89]	-0.74+	[-1.50, 0.02]	-0.94+	[-1.89, 0.02]
	Family high-income	-0.41	[-1.25, 0.44]	-0.47	[-1.10, 0.16]	-0.44	[-1.09, 0.22]	-0.64	[-1.45, 0.17]
	PCG employed	-0.87**	-1.53, -0.21]	-0.50+	[-1.07, 0.07]	-0.75*	[-1.33, -0.17]	-0.43	[-1.08, 0.22]
	Family owns home	-0.39	[-1.03, 0.25]	-0.38	[-0.92, 0.17]	-0.34	[-0.90, 0.22]	-0.59+	[-1.22, 0.03]
	Exposure to violence	0.83	[-0.31, 1.97]	0.64	[-0.40, 1.67]	0.35	[-0.75, 1.46]	0.83	[-0.30, 1.96]
	Large family size	0.12	[-0.50, 0.74]	0.04	[-0.49, 0.56]	-0.17	[-0.71, 0.36]	-0.04	[-0.64, 0.56]
	High-poverty neighborhood = 0	-4.47***	[-5.37, -3.58]	-1.78***	[-0.41, 0.60]	-2.64***	[-3.45, -1.83]	-4.62***	[-5.48, -3.75]
	Constant	-1.07*	[-2.14, 0.47]	0.98	[-0.22, 2.18]	2.12***	[0.95, 3.29]	2.70***	[1.36, 4.04]
	N	805		805		805		805	
	log likelihood	-936.21		-936.21		-936.21		-936.21	
	Pseudo R2	0.27		0.27		0.27		0.27	

*Note*: 95% confidence intervals in brackets; *High-poverty neighborhoods* are census tracts with >20 percent poverty rate. Younger cohort was born between 1994 and 1996; older cohort consists of children that were generally 9, 12, and 15 from 1994–96 (see Table 1). Due to small cell sizes, and because they are not the focus of this analysis, we exclude "other race" (n = 33) in multinomial regression models predicting membership in observed trajectory groups. We used factor analysis to calculate the baseline family composite score, which summarizes answers to six correlated interview questions about family personal and institutional troubles into a single measure with higher values indicating greater troubles (mean = 0, sd = 1). *Exposure to violence* is a dichotomous measure indicating exposure at baseline. *High-income* is a dichotomous indicator denoting household income greater than 50,000 dollars in 1995. *PCG* refers to parental caregiver. *Large family size* is a binary measure denoting a household size with five or more members.

^a^ DA = durably advantaged; PP = persistent poverty; IP = increasing poverty; MP = marginal poverty; DP = decreasing poverty.

+ p<0.1

* p<0.05

** p<0.01

*** p<0.001.

The relative likelihood of following any non-DA trajectory compared with following the durably advantaged trajectory is substantially higher for Black individuals relative to whites (reference category), as indicated by the significant negative main effects across all trajectories. Hispanic children are also more likely to follow a non-DA trajectory relative to white children, though the relative risk is substantially lower compared to Black children, and the likelihood of membership is fairly similar for all non-DA groups. While Black sample members, compared to whites, are associated with a higher risk of membership in all high-poverty trajectory types relative to those in durably advantaged low-poverty trajectories, the odds of following the persistently high-poverty neighborhood trajectory are substantially and significantly greater. Put together, and mirroring the descriptive results from Fig 3, we observe the sheer persistence of advantaged residential contexts in which white sample children find themselves. On the other hand, there tends to be far greater heterogeneity and change in the residential pathways that Black and Hispanic children follow.

Parental employment in childhood significantly lowers the risk of membership in the PP and MP trajectories, relative to membership in the DA trajectory. On the other hand, having a parent that attained a college degree attenuates the risk for membership all non-DA groups. Childhood family income does not significantly predict membership in any of the trajectories, demonstrating that not all baseline family SES factors lower the risk of membership in non-DA groups, relative to the DA group. However, in models that separately interact race with cohort and family income, the risk of following the IP, MP, and DP (trend level significance) trajectories, relative to following the durably advantaged trajectory, is significantly lowered for Black children from high-earning childhood households (see Appendix Table S3 in S1 Appendix).

Finally, we also observe evidence of period effects that predict membership in poverty trajectory groups. The relative risk of following the persistently poor (PP) or marginal poverty (MP) trajectories is significantly lower for the younger birth cohort relative to the older cohort across similar ages, and slightly and marginally lower for following decreasing poverty trajectories. This underscores, on a conceptual and empirical level, the importance of disaggregating between children growing up during different periods since specific macrolevel historical context may shape their trajectories differently depending on when they came of age [37,40].

## The consistency of white advantage and the heterogeneity of nonwhite disadvantage

Our trajectory analyses uncover both stability and change in exposure to neighborhood poverty by race and birth cohort. Advancing beyond prior neighborhood-focused research that typically evaluates an average neighborhood context, our study provides a nuanced portrait of individuals’ cumulative residential environments that incorporates the sequence and timing of exposure. Over time, and between children coming of age during different periods, we find strikingly stable homogeneity of white advantage and dynamic heterogeneity of nonwhite disadvantage in their residential environments. An important finding from our study is that heterogeneity in neighborhood poverty impacts Black children the most, and, to a somewhat lesser degree, Hispanic children. Our results imply that researchers and practitioners should dig deeper into the correlates and explanations for the differences within Black and Hispanic children. Our group-based trajectory method allows us to conclude that typical methodological approaches that capture of an average effect, such as growth curve models or transition matrices, miss this important distinction of within-group heterogeneity.

Our study first examined the different neighborhood poverty trajectories that individuals follow as they age from childhood to young adulthood and asked whether they vary within and between racial/ethnic groups. Does childhood neighborhood context follow individuals as they age through different life stages? For white children, our results suggest that it does. Findings from our group-based trajectory analyses further reveal that most white children travel along the same advantaged path regardless of when they came of age.

These findings arguably demonstrate another way in which white advantage persists in contemporary society. While there are differences in their absolute levels of exposure to neighborhood sociodemographic contexts, white sample members from both cohorts, younger and older, overwhelmingly follow durably low-poverty residential pathways that continue through adulthood, regardless of baseline family factors during childhood. Thus, while prior work examining differences in neighborhood contexts often observes from the perspective of nonwhite children’s disadvantaged residential contexts, our results suggest that another useful frame for understanding longstanding disparities in residential environments is through a lens that focuses on the persistence and resiliency of white advantage.

On the other hand, Black and Hispanic trajectories are characterized by considerable heterogeneity. Both within and between racial/ethnic groups, Black and Hispanic children follow multiple residential pathways. Interestingly, Hispanics are overrepresented in the residential trajectory with a relatively lower risk of poverty than all groups except for the durably advantaged trajectory. Hispanics from the younger cohort tend to follow lower-poverty trajectories than older Hispanics, offering some evidence of period effects.

While Black sample members are overrepresented in the residential trajectory characterized by increasing propensity of residing in high-poverty neighborhoods from childhood to adulthood, they are also overrepresented in the trajectory that experiences *decreasing* risk of poverty exposure. Moreover, both Black and Hispanic sample members are represented in all trajectory groups. These collective results suggest important differentiation in the residential experiences of nonwhite children over the life course that past work has overlooked. Our findings call for future work to view the residential pathways of nonwhite children as more nuanced or heterogeneous than prior research acknowledges, beyond the persistent poverty pathways that confirm durable racial inequality compared to white individuals.

Our study further explored potential cohort differentiation in residential trajectories of exposure to neighborhood disadvantage. By incorporating an inter-cohort analysis, our study foregrounds how macrolevel forces differentially influence residential poverty trajectories of children that grew up and came of age during different periods, a form of cohort differentiation due to social change. Our results indicate differences in the risk of neighborhood poverty exposure between cohorts, with the younger birth cohort generally experiencing the most advantaged residential pathways. Identifying empirically the precise social changes that account for cohort differentials in trajectories is beyond the scope of this paper, but they deserve attention in future research. For example, did the Great Recession and its ensuing foreclosure crisis, which coincided with early adulthood for the older cohort, result in an economic fallout that was worse and more enduring for older cohort members relative to the younger cohort members who were experiencing early adolescence during this same period? Did the deconcentration of poverty in Chicago neighborhoods since the 2000s result in younger cohort members generally being more exposed to lower poverty neighborhoods overall? Can the dynamic residential trajectories for Hispanic and Black children be explained by more widespread gentrification in Chicago neighborhoods since the mid-2000s? In the case of gentrification, an individual’s exposure to neighborhood poverty could change as the result of higher-SES households moving into their existing neighborhoods or nonwhite gentrification that sees Black and Hispanic households moving to gentrifying neighborhoods. Although these are important questions, by conditioning our analyses on classic background factors of childhood exposure, we can nonetheless reasonably conclude that broader social changes rather than cohort compositional features are the main driver of differences in poverty trajectory groups across younger versus older cohorts.

Our study leaves open several other questions as well, which we hope will motivate future work. Our study focuses on individuals’ varied sequence of exposure to one economic dimension of residential context: neighborhood poverty. Future research should examine the various pathways of exposure that children follow in terms of other sociodemographic and ecological features, such as racial isolation or environmental toxins like lead or airborne pollution. Future research could also use the identified neighborhood trajectories as predictors and examine the effects of following various residential pathways on later-life outcomes [64]. Another line of inquiry might investigate when exposure to various trajectories matter most for predicting individual outcomes in later adulthood and whether this has changed over time. Due to data limitations, our study is limited to a study frame that follows children through early adulthood. Researchers with information on residential histories might examine trajectories over a longer period of time or during different life stages. Moreover, we rely on data that follows children from Chicago in the 1990s. Other work should examine whether residential patterns hold in other cities within and outside the U.S.

In sum, that we find both durability (of white advantage) and dynamism (in nonwhite pathways) suggests the need to change the way we frame residential processes of stratification over time. That we find differences between cohorts, controlling for their baseline composition, suggests that we pay greater attention to the ways in which individual pathways intersect with broader population dynamics and macrolevel change, a classic theme of demography and yet one which is often unrealized in longitudinal studies of neighborhood stratification. While much past work highlights overall disparities between racial/ethnic groups in their exposure to neighborhood environments, our findings highlight the theoretical and empirical need to also consider the dynamic heterogeneity that exists within racial/ethnic groups and between periods.

## Supporting information

S1 Appendix Contains all supporting appendix tables and figures. (DOCX)
