# Supplementary material for: Diverging trajectories of neighborhood disadvantage by race and birth cohort from childhood through young adulthood
Source: PLoS One. 2023 Apr 19;18(4):e0283641. doi: 10.1371/journal.pone.0283641 (PMC10115268; doi:10.1371/journal.pone.0283641)
Supplement: S1 Appendix — (DOCX) [file pone.0283641.s001.docx]

# Supplementary Material S1

**Appendix Table S1.** Model fit statistics, for group-based trajectory prevalence models (high-poverty) with 1-7 groups (N=838)

| No. of groups | Averaged Predicted Probability (APP) of Assignment into Each Group | | | | | | | BIC (persons) | BIC (person-years) | % in smallest group |
| --- | --- | --- | --- | --- | --- | --- | --- | --- | --- | --- |
|  | 1 | 2 | 3 | 4 | 5 | 6 | 7 |  |  |  |
| 1 | 1.000 |  |  |  |  |  |  | -14711.5 | -14714.5 | 100.0% |
| 2 | 0.986 | 0.986 |  |  |  |  |  | -8264.2 | -8292.9 | 43.0% |
| 3 | 0.986 | 0.974 | 0.983 |  |  |  |  | -7398.4 | -7452.9 | 25.1% |
| 4 | 0.965 | 0.961 | 0.976 | 0.984 |  |  |  | -6918.7 | -6998.8 | 19.8% |
| **5** | **0.976** | **0.944** | **0.948** | **0.957** | **0.963** |  |  | **-6724.4** | **-6830.2** | **18.3%** |
| 6 | 0.958 | 0.976 | 0.949 | 0.946 | 0.938 | 0.933 |  | -6518.9 | -6650.4 | 14.7% |
| 7 | 0.980 | 0.952 | 0.935 | 0.923 | 0.918 | 0.914 | 0.924 | -6441.7 | -6598.9 | 7.5% |

*Note*: Results drawn from prevalence trajectory models estimating group membership based on the risk of residing in high-poverty neighborhoods (> 20 percent) over time. Models 2-7 use all cubic age terms and one linear term. The table displays the average posterior probabilities of trajectory group membership and Bayesian Information Criterion (BIC) statistics of model. The selected model is in bold. Models include sample members sufficient values of risk variables in cumulative observed waves. N=838 person observations and 17232 person-year observations used in the trajectory analysis.

**Appendix Table S2.** Comparison of Measures in Analysis File vs. Full Geocoded Sample

|  |  | Analysis Sample (N=838) | Full Sample (N=1057) |
| --- | --- | --- | --- |
| Individual measures | |  |  |
|  | White | 0.192 | 0.190 |
|  | Black | 0.371 | 0.365 |
|  | Hispanic | 0.397 | 0.403 |
|  | Other race | 0.039 | 0.042 |
|  | Younger cohort | 0.325 | 0.358 |
|  | Older cohort | 0.675 | 0.642 |
|  | Female | 0.511 | 0.513 |
|  | Male | 0.489 | 0.487 |
| Baseline family measures | |  |  |
|  | Composite family score | 0.003 | 0.000 |
|  | Parent college degree (BA) | 0.154 | 0.147 |
|  | Family high-income | 0.277 | 0.263 |
|  | PCG employed | 0.673 | 0.658 |
|  | Family owns home | 0.469 | 0.445 |
|  | Exposure to violence | 0.091 | 0.098 |
|  | Large family size | 0.560 | 0.574 |
|  | High-poverty neighborhood=0 | 0.549 | 0.550 |
|  | High-poverty neighborhood=1 | 0.451 | 0.450 |

Note: This table compares the distribution and means of key measures in the analysis sample to the full geocoded sample prior to analytic filters. While the older cohort is represented slightly more in the analysis file, the two samples show substantial similarity across all values.

**Appendix Table S3.** Multinomial logistic regression of risk factors on high-poverty neighborhood trajectories (baseline outcome = durably advantaged (DA))^a^

|  |  | PP |  | IP |  | MP |  | DP |  |
| --- | --- | --- | --- | --- | --- | --- | --- | --- | --- |
|  |  | Log odds | [95% CI] | Log odds | [95% CI] | Log odds | [95% CI] | Log odds | [95% CI] |
| *Ref. category: DA* | |  |  |  |  |  |  |  |  |
| Individual factors | |  |  |  |  |  |  |  |  |
|  | White | (ref) |  | (ref) |  | (ref) |  | (ref) |  |
|  | Black | 4.63*** | [2.81,6.44] | 4.22*** | [2.90,5.55] | 2.81*** | [1.59,4.04] | 2.90*** | [1.31,4.50] |
|  | Hispanic | 1.76* | [0.11,3.42] | 1.59** | [0.51,2.67] | 1.26** | [0.35,2.16] | 1.52* | [0.16,2.88] |
|  | Older (9/12/15) Cohort | (ref) |  | (ref) |  | (ref) |  | (ref) |  |
|  | Younger (0) Cohort | -14.99 | [-1650.8,1620.8] | 0.85 | [-0.29,1.98] | -16.5 | [-1882.6,1849.6] | -2.01 | [-4.42,0.39] |
|  | Female | (ref) |  | (ref) |  | (ref) |  | (ref) |  |
|  | Male | 0.15 | [-0.45,0.75] | 0.14 | [-0.37,0.65] | 0.11 | [-0.41,0.63] | -0.34 | [-0.93,0.25] |
| Baseline measures | |  |  |  |  |  |  |  |  |
|  | Composite family score | 0.07 | [-0.24,0.38] | 0.04 | [-0.24,0.32] | 0.21 | [-0.05,0.47] | -0.14 | [-0.48,0.20] |
|  | Parent BA | -1.44* | [-2.55,-0.32] | -1.60*** | [-2.40,-0.80] | -0.62 | [-1.38,0.14] | -0.93+ | [-1.89,0.04] |
|  | Family high-income | 0.56 | [-1.66,2.78] | 0.2 | [-0.99,1.40] | 0.12 | [-1.03,1.27] | 0.27 | [-1.51,2.04] |
|  | PCG employed | -0.88** | [-1.55,-0.22] | -0.49+ | [-1.07,0.09] | -0.78** | [-1.37,-0.19] | -0.44 | [-1.11,0.22] |
|  | Owns home | -0.45 | [-1.11,0.21] | -0.50+ | [-1.07,0.06] | -0.38 | [-0.95,0.19] | -0.58+ | [-1.23,0.07] |
|  | Exposure to violence | 0.84 | [-0.32,2.00] | 0.6 | [-0.45,1.65] | 0.3 | [-0.83,1.42] | 0.88 | [-0.27,2.04] |
|  | Large family size | 0.06 | [-0.58,0.69] | 0.04 | [-0.49,0.57] | -0.23 | [-0.77,0.31] | -0.27 | [-0.89,0.35] |
|  | High-poverty neighborhood=0 | -4.49*** | [-5.41,-3.58] | -1.83*** | [-2.67,-0.98] | -2.69*** | [-3.53,-1.86] | -4.68*** | [-5.58,-3.78] |
| Interactions | |  |  |  |  |  |  |  |  |
|  | Black x Cohort 0 | 13.39 | [-1622.4,1649.2] | -0.75 | [-2.35,0.85] | 15.68 | [-1850.5,1881.8] | 2.82* | [0.11,5.53] |
|  | Hispanic x Cohort 0 | 13.19 | [-1622.6,1649.0] | -0.73 | [-2.08,0.63] | 15.47 | [-1850.7,1881.6] | 0.48 | [-2.07,3.03] |
|  | Black x High-income | -1.73 | [-4.28,0.82] | -1.78* | [-3.44,-0.13] | -1.99* | [-3.72,-0.26] | -2.22+ | [-4.45,0.01] |
|  | Hispanic x High-income | -1.93 | [-4.66,0.81] | -0.73 | [-2.23,0.77] | -0.37 | [-1.75,1.02] | -0.99 | [-3.06,1.08] |
|  | Constant | 1.79+ | [-0.04,3.63] | 0.98 | [-0.22, 2.18] | 2.13** | [0.85,3.40] | 2.63** | [1.04,4.23] |
|  | N | 805 |  | 805 |  | 805 |  | 805 |  |
|  | log likelihood | -912.91 |  | -912.91 |  | -912.91 |  | -912.91 |  |
|  | Pseudo R2 | 0.29 |  | 0.29 |  | 0.29 |  | 0.29 |  |

*Note*: 95% confidence intervals in brackets; *High-poverty neighborhoods* are census tracts with >20 percent poverty rate. Younger cohort was born between 1994 and 1996; older cohort consists of children that were generally 9, 12, and 15 from 1994-96 (see Table 1). Due to small cell sizes, and because they are not the focus of this analysis, we exclude "other race" (n=33) in multinomial regression models predicting membership in observed trajectory groups. *PCG* refers to parental caregiver.

^a^ DA = durably advantaged; PP = persistent poverty; IP = increasing poverty; MP = marginal poverty; DP = decreasing poverty;

+ p<0.1, * p<0.05, ** p<0.01, *** p<0.001

**Appendix Figure S1.** Predicted Probability of Membership in Observed High-Poverty Residential Trajectory, by Race and Cohort


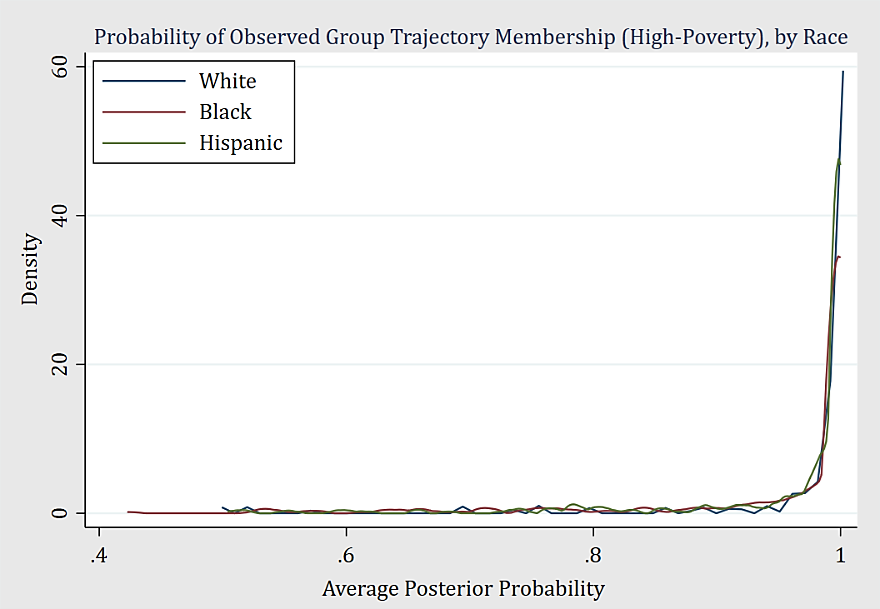

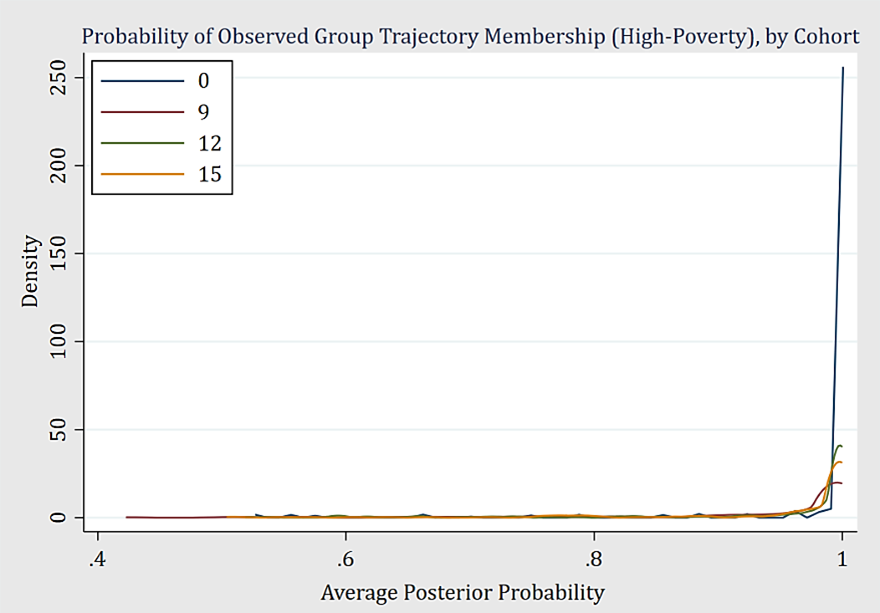


Note: Kernel density distributions, by race (left) and 4-category cohort (right), drawn from a group-based trajectory prevalence model (5-group) with cubic age terms identifying trajectories of high-poverty neighborhood exposure. The 9-, 12-, and 15-year-old groups represent the “older” cohort.

Appendix Figure A1 displays kernel density plots of the predicted probability of membership into the observed trajectory for all children included in group-based trajectory prevalence models (GBTM) for residence in high-poverty neighborhoods from 1995 to 2018. Our GBTM model predicts membership well for both cohorts and for white, Black, and Hispanic sample members. The average predicted probability is well above the 0.70 minimum threshold recommended by Nagin (2005) in considering best-model fit.

References:

Nagin D. Group-based Modeling of Development . Cambridge, MA: Harvard University Press; 2005.

**Appendix Figure S2.** Comparison of Mean Neighborhood Poverty using Unweighted (left) and Weighted (right) Samples (by Race and Cohort, 1995-2018)


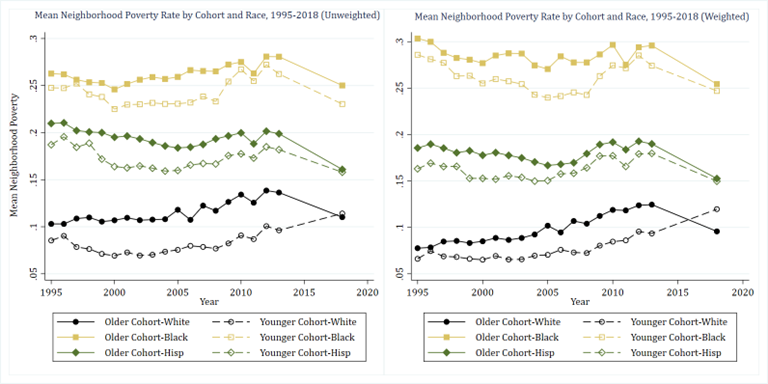


Note: Weighted analysis uses design stratification and attrition weights. Information on neighborhood poverty is derived from decennial census data and 5-year American Community Survey estimates. The main difference between the weighted and unweighted samples is that the neighborhood poverty rate for Black individuals is slightly higher than the rates in the unweighted sample. The trend lines themselves, however, are very similar and the main substantive findings hold. That Black individuals have somewhat higher mean neighborhood poverty levels in the weighted sample is not surprising given that poor Black individual were less likely to be in final sample (as with most surveys), which is why they are upweighted relative to a non-weighted sample. Because our main quantity of interest is the trajectories themselves within our sample rather than absolute neighborhood poverty levels, this difference does not influence our findings.

**Appendix Figure S3.** Mean Neighborhood Poverty Rate by Trajectory Group


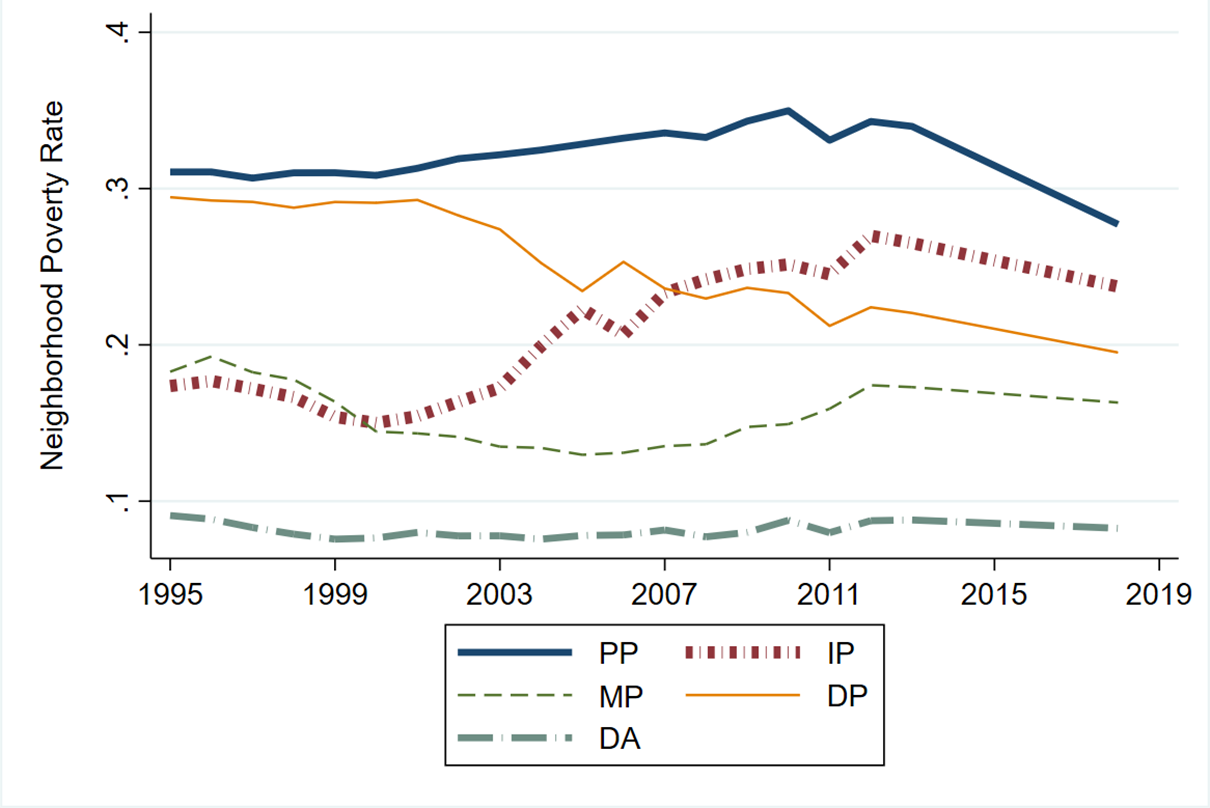


Note: This figure plots the mean neighborhood poverty rate for sample members from 1995 to 2019. Based on their probability of membership, we assign sample members to one of the five trajectory groups that we identify during group-based trajectory (GBT) analysis (See Figure 2). The patterns that emerge when plotting the raw neighborhood poverty levels (a continuous outcome) mirrors what we find in our GBT prevalence model which observes a binary outcome for high neighborhood poverty.

1) PP = persistent poverty (18.5%); 2) IP = increasing poverty (18.7%); 3) MP = marginal poverty (17.1%); 4) DP = decreasing poverty (18.9%); 5) DA = durably advantaged (26.8%).

**Appendix Figure S4.** Ratio of Observed to Expected (if equally distributed) racial composition in high-poverty trajectory groups

**
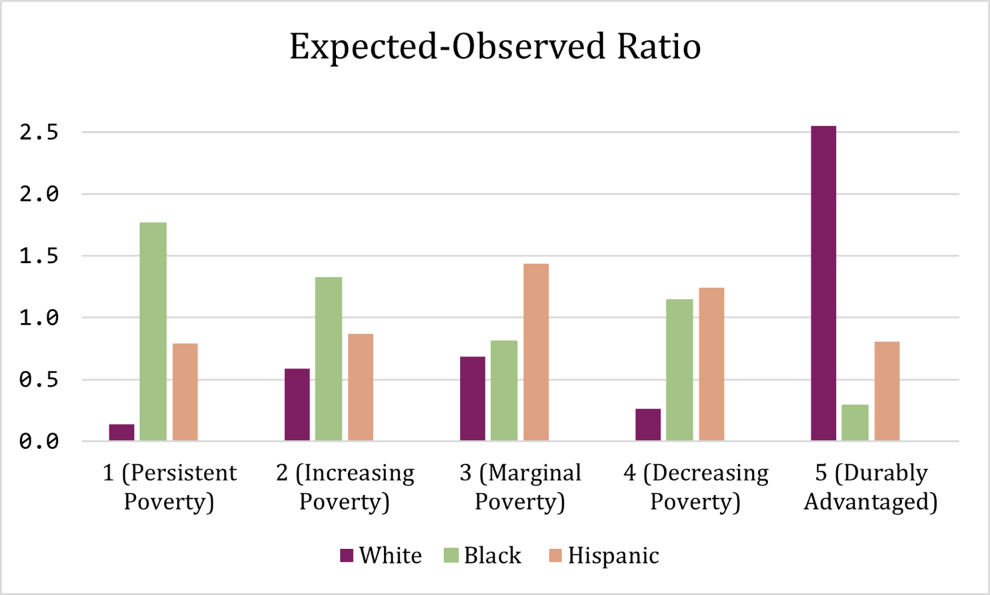
**

*Note*: Expected racial composition is based on the overall distribution of racial/ethnic groups in the sample used for group-based trajectory models. If sample members were evenly distributed by race into the trajectory groups, we would expect the distribution for each of the five trajectory types to mirror the overall distribution of the sample.

**Appendix Figure S5**: Ratio of Observed to Expected (if equally distributed) cohort composition in high-poverty trajectory groups

*
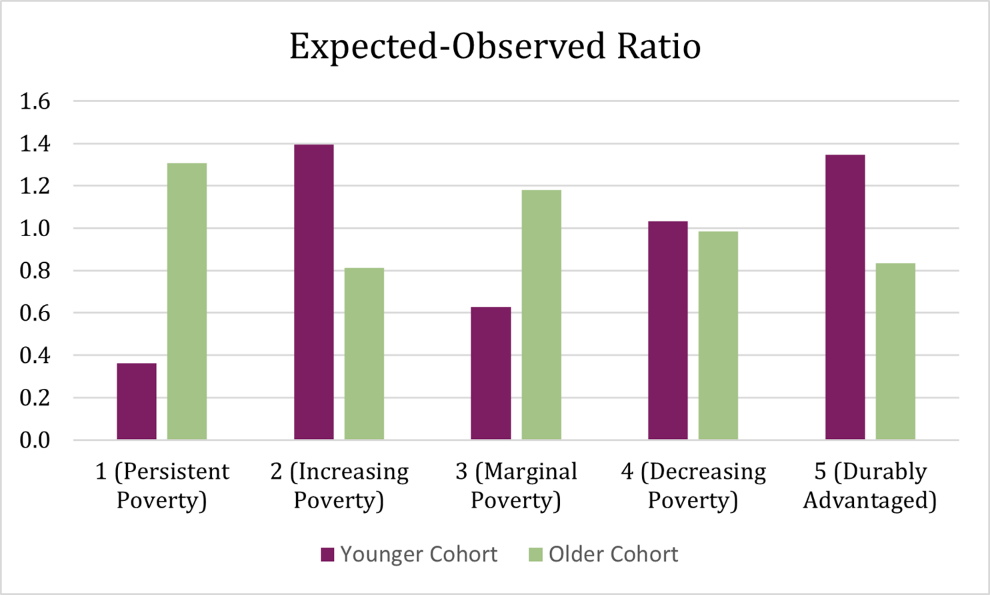
*

*Note*: Expected cohort composition is based on the overall distribution of cohort groups in the sample used for group-based trajectory models. If sample members were evenly distributed by race into the trajectory groups, we would expect the distribution for each of the five trajectory types to mirror the overall distribution of the sample.
